# Attributable mortality and excess length of stay associated with third-generation cephalosporin-resistant Enterobacterales bloodstream infections: a prospective cohort study in Suva, Fiji

**DOI:** 10.1016/j.jgar.2022.06.016

**Published:** 2022-09

**Authors:** Michael J. Loftus, Tracey E.M.W. Young-Sharma, Sue J. Lee, Shitanjni Wati, Gnei Z. Badoordeen, Luke V. Blakeway, Sally M.H. Byers, Allen C. Cheng, Ben S. Cooper, Hugh Cottingham, Adam W.J. Jenney, Jane Hawkey, Nenad Macesic, Ravi Naidu, Amitesh Prasad, Vinita Prasad, Litia Tudravu, Timoci Vakatawa, Elke van Gorp, Jessica A. Wisniewski, Eric Rafai, Anton Y. Peleg, Andrew J. Stewardson

**Affiliations:** a Department of Infectious Diseases, The Alfred Hospital and Central Clinical School, Monash University, Melbourne, Australia; b Colonial War Memorial Hospital, Suva, Fiji; c School of Public Health and Preventive Medicine, Monash University, Melbourne, Australia; d Centre for Tropical Medicine and Global Health, Nuffield Department of Medicine, University of Oxford, Oxford, United Kingdom; e Mahidol-Oxford Tropical Medicine Research Unit, Mahidol University, Bangkok, Thailand; f Fiji National University, Suva, Fiji; g Centre to Impact AMR, Monash University, Melbourne, Australia; h Fiji Ministry of Health and Medical Services, Suva, Fiji; i Infection and Immunity Program, Monash Biomedicine Discovery Institute, Department of Microbiology, Monash University, Clayton, Australia

**Keywords:** Bloodstream infection, Enterobacterales, Mortality, Antimicrobial resistance, Fiji

## Abstract

**Objectives:**

There are scant primary clinical data on antimicrobial resistance (AMR) burden from low- and middle-income countries (LMICs). We adapted recent World Health Organization methodology to measure the effect of third-generation cephalosporin resistance (3GC-R) on mortality and excess length of hospital stay in Fiji.

**Methods:**

We conducted a prospective cohort study of inpatients with Enterobacterales bloodstream infections (BSIs) at Colonial War Memorial Hospital, Suva. We used cause-specific Cox proportional hazards models to estimate the effect of 3GC-R on the daily risk (hazard) of in-hospital mortality and being discharged alive (competing risks), and we used multistate modelling to estimate the excess length of hospital stay.

**Results:**

From July 2020 to February 2021 we identified 162 consecutive Enterobacterales BSIs; 3GC-R was present in 66 (40.7%). Crude mortality for patients with 3GC-susceptible and 3GC-R BSIs was 16.7% (16/96) and 30.3% (20/66), respectively. 3GC-R was not associated with the in-hospital mortality hazard rate (adjusted hazard ratio [aHR] 1.13, 95% confidence interval [CI] 0.51–2.53) or being discharged alive (aHR 0.99, 95% CI 0.65–1.50), whereas Charlson comorbidity index score (aHR 1.62, 95% CI 1.36–1.93) and Pitt bacteraemia score (aHR 3.57, 95% CI 1.31–9.71) were both associated with an increased hazard rate of in-hospital mortality. 3GC-R was associated with an increased length of stay of 2.6 days (95% CI 2.5–2.8). 3GC-R was more common among hospital-associated infections, but genomics did not identify clonal transmission.

**Conclusion:**

Patients with Enterobacterales BSIs in Fiji had high mortality. There were high rates of 3GC-R, which was associated with increased hospital length of stay but not with in-hospital mortality.

## Highlights

• Pacific island countries have contributed few data to global AMR burden estimates.
• We assessed the impact of third-generation cephalosporin resistance (3GC-R) in Fiji.
• 3GC-R was common but mortality was high regardless of the resistance profile.
• We found minimal impact by 3GC-R on the adjusted hazard of in-hospital mortality.
• 3GC-R infections were associated with an increased length of hospital stay.

## 1 Introduction

Antimicrobial resistance (AMR) represents a serious threat to public health worldwide, as patients with AMR infections experience higher crude mortality and increased length of hospital stay and also incur greater healthcare costs compared to patients with susceptible infections [1]. Extended-spectrum beta-lactamases (ESBLs), enzymes conferring resistance to most beta-lactam antibiotics including third-generation cephalosporins (3GC), are of particular concern given their increasing frequency globally [2] and their ability to easily transmit between organisms via plasmids [3].

The greatest burden of infectious diseases (including AMR) falls disproportionately on low- and middle-income countries (LMICs). This is driven by factors relating to sanitation, reduced access to healthcare, lower availability of diagnostic testing, and fewer regulations of antimicrobial use [4], [5], [6]. Despite a pressing need, there is presently little high-quality data on the burden of AMR from LMICs [7], including from the Pacific Islands region [8]. Precisely quantifying the effect of AMR in LMICs is important to allow appropriate investment in strategies to address AMR amongst numerous competing budget priorities.

To assist global efforts to monitor the burden of AMR, especially within LMICs, the World Health Organization (WHO) recently established standardised methodology for assessing the attributable mortality of AMR bloodstream infections (BSIs) [9]. BSIs were chosen as they are the most life-threatening infections, are unambiguously clinically significant, and have been shown to be responsible for the majority of the burden of AMR [10]. The WHO document focuses only on methicillin-resistant *Staphylococcus aureus* and ESBL-producing *Escherichia coli* but anticipates researchers may also incorporate other AMR pathogens relevant to their local context.

We report on our adaptation of this WHO methodology in Fiji, a tropical middle-income country in the Pacific Islands region, to assess the attributable mortality of 3GC resistant (3GC-R) Enterobacterales BSIs at a tertiary hospital. We have also calculated the excess length of hospital stay attributable to AMR, as another key marker of the burden posed by resistant infections.

## 2 Methods

### 2.1 Study design

We conducted a prospective cohort study of consecutive Enterobacterales BSIs among inpatients at the Colonial War Memorial Hospital (CWMH) in Suva, over eight months from July 2020 through February 2021. The primary exposure of interest was 3GC-R, and the primary outcomes were in-hospital mortality and length of hospital stay. As dying in hospital and being discharged alive are competing outcomes, we modelled the effect of 3GC-R on both.

### 2.2 Setting

CWMH is a 500-bed tertiary hospital and the largest healthcare facility in Fiji. It serves a local catchment population of almost 400 000 people and is the national referral centre for Fiji's other two divisional hospitals. It has adult, paediatric, and neonatal intensive care units (ICUs) and offers specialty services including cardiology, nephrology, orthopaedics, plastic surgery, urology, and neurosurgery.

### 2.3 Population

Patients with an Enterobacterales BSI were eligible if they were admitted to CWMH at the time of or up to 48 hours after collection of their index blood culture. Patients were to be excluded if they were not being treated with curative intent for the entire calendar day on which the index blood culture was collected. Patients could be included in the study multiple times if they met inclusion criteria during separate admissions.

### 2.4 Patient identification

We identified potential patients by screening new positive blood culture results each day. Ward-based data collectors then determined whether patients with Enterobacterales BSI were eligible. The medical records of eligible patients were reviewed as soon as practicable.

### 2.5 Data collection

Study data were collected and managed using REDCap electronic data capture tools hosted at Monash University, Australia [11]. Data collection occurred at two time-points: on inclusion and after discharge. Clinical and antibiotic data were collected at the initial chart review. We used WHO definitions [9] unless specified otherwise below and outlined in greater detail in the Supplementary Table S1. Clinical data included patient age, sex, admission date, admission source, admission diagnosis, admitting specialty, age-adjusted Charlson comorbidity index score, Pitt bacteraemia score and high-risk quick Sequential Organ Failure Assessment (qSOFA) score at bacteraemia onset, and whether the patient had any immunosuppression, ICU admission, surgical procedure, or antibiotic exposure preceding their bacteraemia. We augmented the WHO GLASS protocol to also collect the likely anatomical source of infection and the epidemiological attribution of their bacteraemia, categorised as hospital acquired, community acquired, or healthcare associated [12].

We recorded all antibiotics received from day 0 to 2 inclusive (‘initial antibiotic therapy’; day 0 = day of index positive blood culture) and the antibiotic susceptibility testing (AST) results as reported by the Suva lab. We determined whether patients had received ‘active’ initial antibiotic therapy. ‘Active’ therapy was defined as receiving at least one antimicrobial to which the organism was susceptible according to confirmatory AST. Inactive therapy was defined as receipt of antimicrobials that tested nonsusceptible or would be expected to not have activity against Enterobacterales (e.g., cloxacillin or vancomycin). A patient's therapy status was recorded as ‘unknown’ if AST data were missing for at least one potentially effective antimicrobial they received. Ceftriaxone was deemed to be ineffective against both Enterobacter spp. and *Klebsiella aerogenes*, regardless of phenotypic susceptibility, because of their propensity to express chromosomal AmpC [13].

Data on patients’ discharge date and status were collected retrospectively after they had either been discharged or died. Patients’ clinical management was entirely at the discretion of their CWMH clinicians, with no input from the research team.

### 2.6 Laboratory testing

Initial culture, identification, and AST of bacterial isolates were performed by the CWMH laboratory as per their standard procedures. Blood cultures were incubated using the BACT/ALERT system (bioMérieux, Marcy-Étoile, France). Positive cultures with Gram-negative organisms were plated onto 5% sheep blood agar, chocolate agar, and MacConkey agar. Identification and AST (including assessment of ESBL production) was performed using the VITEK-2 GP ID and VITEK-2 AST cards (bioMérieux, Marcy-Étoile, France; version 9.01), respectively. In the rare instance when VITEK was unavailable, the Microbact Gram-negative system (Oxoid) was used for identification, disc diffusion was used for AST, and cefotaxime and ceftazidime discs with clavulanate were used to assess for ESBL production. Clinical and Laboratory Standards Institute (CLSI) breakpoints were used [13].

Where possible, the index isolate from each patient was subcultured, stored at −80°C, and shipped to Melbourne, Australia for confirmatory identification using MALDI-TOF MS (Bruker, Hanau, Germany) and confirmatory AST using VITEK-2 AST cards (bioMérieux, version 8.01). Repeat testing was performed on any isolates with discrepant results, and if a difference remained, the Melbourne result was used.

Isolates were classified as 3GC-R if there was evidence of either ESBL production or nonsusceptibility to at least one third-generation (ceftriaxone, ceftazidime) or fourth-generation (cefepime) cephalosporin. As mentioned above, all Enterobacter spp. and *K. aerogenes* isolates were deemed 3GC-R regardless of reported phenotype. The remaining isolates were classified as 3GC susceptible (3GC-S).

### 2.7 Genomic analyses

We performed whole genome sequencing (WGS) on a representative subset of bacterial isolates, stratified by bacterial species and month of isolation. All bacterial isolates were routinely grown on Heart Infusion agar (Oxoid) for 16 h at 37°C, inoculated into 3 mL Heart Infusion broth (Oxoid), and grown for a further 16 h at 37°C with orbital shaking at 150 rpm. Genomic DNA was extracted from 300 µL of liquid bacterial culture using the GenFind V3 Reagent Kit (Beckman Coulter) as per manufacturer's instructions. Libraries were prepared using the Nextera Flex DNA Library Prep Kit (Illumina), and 150 bp paired-end sequencing was performed on the NovaSeq 6000 system (Illumina). Genomes were assembled using Unicycler (v.0.4.9) [14]. Multi-locus sequence types (STs) were identified using mlst (https://github.com/tseemann/mlst).

For the genomes of the most prevalent ST, we determined relatedness by calculating pairwise single nucleotide variation (SNV) distances between all isolates. Genomes were assembled using SKESA v.2.3 [15] using default parameters. Each pair of genome assemblies was compared using Catpac (https://github.com/rrwick/Catpac) to determine the number of SNVs between pairs. Pairs with ≤5 SNVs per mbp between them were considered likely candidates for strain transmission, as defined by Gorrie et al. [16].

### 2.8 Statistical analysis

Our sample size estimate was based on an expected in-hospital mortality rate of 45% among patients with 3GC-R Enterobacterales BSIs, and 20% among those with susceptible infections. In the absence of known mortality rates from Fiji, we used published mortality rates for 3GC-S and 3GC-R Enterobacterales BSIs from another tropical LMIC [17]. Assuming an alpha value of .05 and 80% power, we required 52 patients in each group to test the hypothesis that mortality is higher among 3GC-R BSIs than among susceptible ones.

We used Cox proportional hazards models to quantify the effect of antimicrobial resistance on mortality. To account for the fact that in-hospital mortality and being discharged alive are competing events, we performed cause-specific Cox proportional hazards models for both of these outcomes [9,18]. Infections, rather than patients, were used as the unit of analysis. In addition to 3GC susceptibility status, multivariable models included three variables selected using a directed acyclic graph created with DAGitty [19]: Charlson comorbidity index score, Pitt bacteraemia score, and recent hospitalisation (within 90 days) (Supplementary Fig. S1). Pitt bacteraemia score was included as a categorical variable (scores of 0–1, 2–3, and ≥4) [20]. All models used BSI onset as time zero and were adjusted for time from admission to infection. We checked the proportional hazards assumption using Schoenfeld residuals and visual inspection of log-log plots. Nonproportional hazards were corrected using stratification. To assess the effect of initial treatment on mortality, we created another set of Cox proportional hazards models but with receipt of active initial therapy as the exposure of interest.

We estimated the excess length of stay attributable to 3GC-R using a multistate model with four states (admission, resistant, susceptible, and discharged/died) [21]. The expected length of stay was estimated for each day in the resistant and susceptible states using Aalen-Johansen estimators for transition probabilities. Difference in the length of stay was calculated between those with 3GC-R infections and those with 3GC-S infections, and then a weighted average was estimated using the observed distribution of time to infection. Standard errors and 95% confidence intervals were derived by bootstrapping. In parallel, we also estimated the effect of 3GC-R on length of stay using Cox proportional hazards regression as above, but in this case with a composite all-cause end-of-stay endpoint (either death or discharge alive). This could be interpreted as an indication of the daily hazard of a patient's admission ending.

## 3 Results

### 3.1 Patient characteristics

We included 159 patients with 162 BSI episodes; three patients met inclusion criteria on two separate CWMH admissions. 3GC-R organisms were responsible for 66 BSIs (40.7%). Median patient age was 55 years. We included 20 children (age <18 years) and 10 neonates (age <28 days) (Table 1). The prevalence of diabetes among adults was 62/142 (43.7%). No paediatric patients had diabetes.

**Table 1:** Characteristics of patient cohort

	Overall (n = 162)	3GC-S (n = 96)	3GC-R (n = 66)
**Demographics**			
Male, n (%)	71 (43.8%)	39 (40.6%)	32 (48.5%)
Median age (IQR)	55.4 (38.1, 67.7)	54.3 (36.1, 65.8)	56.1 (40.3, 70.0)
Paediatric (<18 y), n (%)	20 (12.3%)	12 (12.5%)	8 (12.1%)
Source of admission (n = 161), n (%)			
Home	114 (70.8%)	67 (70.5%)	47 (71.2%)
Transfer from another facility	39 (24.2%)	24 (25.3%)	15 (22.7%)
Birth	8 (5.0%)	4 (4.2%)	4 (6.1%)
Ward (when index BC collected), n (%)			
Emergency department	78 (48.1%)	57 (59.4%)	21 (31.8%)
Medical or surgical ward	29 (17.9%)	7 (7.3%)	22 (33.3%)
Inpatient at another facility	21 (13.0%)	14 (14.6%)	7 (10.6%)
Intensive care unit^a^	16 (9.8%)	3 (3.1%)	13 (19.7%)
Maternity ward	10 (6.2%)	10 (10.4%)	0 (0.0%)
Paediatric ward	5 (3.1%)	3 (3.1%)	2 (3.0%)
Other ward	3 (1.9%)	2 (2.1%)	1 (1.5%)
**Acquisition of BSI (n = 161)**			
Community acquired	107 (66.5%)	79 (83.2%)	28 (42.4%)
Hospital acquired	39 (24.2%)	9 (9.5%)	30 (45.5%)
Healthcare-associated community onset	15 (9.3%)	7 (7.4%)	8 (12.1%)
**Healthcare exposures**			
Antibiotics in 30 days prior (n = 157), n (%)	58 (36.9%)	26 (28.0%)	32 (50.0%)
Hospitalised in 90 days prior, n (%)	36 (22.2%)	17 (17.7%)	19 (28.8%)
Surgery since admission (n = 160), n (%)	17 (10.6%)	5 (5.3%)	12 (18.5%)
Immunosuppressed (n = 159), n (%)	18 (11.3%)	6 (6.5%)	12 (18.2%)
ICU stay prior to BSI (n = 161), n (%)	15 (9.3%)	1 (1.0%)	14 (21.2%)
Median LoS prior to BSI (IQR)	0 (0, 2)	0 (0, 0)	0 (0, 8)
**Clinical risk scores**			
Median age-adjusted Charlson Comorbidity Index score (IQR)	2 (0, 4)	2 (0, 3)	2 (1, 5)
Median Pitt bacteraemia score (IQR)	1 (0, 2)	1 (0, 2)	1 (1, 4)
High-risk qSOFA score (n = 156), n (%)	36 (23.1%)	16 (17.0%)	20 (32.3%)
**Microbiology**			
Organism grown			
*E. coli*	85 (52.5%)	56 (58.3%)	29 (43.9%)
* K. pneumoniae*	48 (29.6%)	25 (26.0%)	23 (34.8%)
Other Enterobacterales^b^	29 (17.9%)	15 (15.6%)	14 (21.2%)
Presumed source of infection			
Primary	98 (60.5%)	64 (66.7%)	34 (51.5%)
Central line associated	7 (4.3%)	0 (0.0%)	7 (10.6%)
Secondary	57 (35.2%)	32 (33.3%)	25 (37.9%)
Anatomic origin of infection (for secondary infections only)			
Urinary tract infection	27 (47%)	15 (47%)	12 (48%)
Skin and soft tissue infection	12 (21%)	8 (25%)	4 (16%)
Other	18 (31%)	9 (28%)	9 (36%)
**Active empiric antibiotics**			
Yes	92 (56.8%)	79 (82.3%)	13 (19.7%)
No	61 (37.7%)	14 (14.6%)	47 (71.2%)
Unknown	9 (5.6%)	3 (3.1%)	6 (9.1%)
**Outcomes**			
Died, n (%)	36 (22.2%)	16 (16.7%)	20 (30.3%)
Median overall LoS (IQR)	10 (6, 17)	8 (5.5, 13)	13 (8, 22)
Median LoS following BSI (IQR)	9 (5, 16)	8 (5.5, 13)	11 (5, 17)
Among survivors (n = 126);	10 (7, 16)	9 (6, 11.5)	13 (8, 17)
Among those who died (n = 36)	4 (1, 12.5)	5 (1, 12.5)	4 (1.5, 12.5)

BC, blood culture; BSI, bloodstream infection; ICU, intensive care unit; IQR, interquartile range; LoS, length of stay; qSOFA, quick Sequential Organ Failure Assessment.

a The ward ‘intensive care unit’ includes patients from any of the adult, paediatric, or neonatal ICUs.

b These 29 ‘Other’ Enterobacterales consisted of: 8 *Proteus mirabilis*; 6 *Enterobacter cloacae*, 4 *Citrobacter koseri*, 3 *Serratia marcescens*, 2 *Morganella morganii*, 2 *Klebsiella aerogenes*, 1 *Enterobacter agglomerans*, 1 *Enterobacter bugandensis*, 1 *Klebsiella variicola*, and 1 *Providencia rettgeri.*

Paediatric cases had a slightly lower proportion of community-acquired infections (11/20, 55.0%) and 3GC-R infections (8/20, 40.0%) while also experiencing lower crude in-patient mortality (2/20, 10.0%) when compared with adults. A full comparison of the paediatric and adult cohorts can be found in Supplementary Table S2.

### 3.2 Mortality

All-cause in-hospital mortality was higher among patients with 3GC-R infections (20/66, 30.3%) compared to those with 3GC-S infections (16/96, 16.7%). While the univariable cause-specific Cox model was consistent with 3GC-R being associated with an elevated hazard of in-hospital mortality, there was substantial uncertainty (aHR 1.67, 95% confidence interval [CI] 0.80–3.49). After adjusting for confounders, the magnitude of this effect diminished substantially (aHR 1.13, 95% CI 0.51–2.53). 3GC-R was not associated with a large change in the hazard of discharge alive, on either univariable or multivariable models (Table 2).

**Table 2:** Effect of third generation cephalosporin resistance and patient factors on in-hospital mortality and discharge alive, using Cox proportional hazards models^a^

	In-hospital mortality	Discharge alive
	Hazard ratio (95% CI)	Hazard ratio (95% CI)
**Univariable model**		
3GC resistance	1.67 (0.80–3.49)	0.75 (0.50–1.12)
**Multivariable model**		
3GC resistance	1.13 (0.51–2.53)	0.99 (0.65–1.50)
Age-adjusted Charlson comorbidity index score	1.62 (1.36–1.93)	0.93 (0.84–1.03)
Pitt bacteraemia score		
0–1	Reference	Reference
2–3	3.57 (1.31–9.71)	0.91 (0.58–1.44)
4+	13.0 (5.21–32.6)	0.15 (0.06–0.37)
Recent hospitalisation	2.34 (1.18–4.65)	0.97 (0.59–1.61)

3GC, third-generation cephalosporin, CI, confidence interval.

a Both models included adjustment for pre-BSI hospital length of stay (days).

In the multivariable models, higher Charlson comorbidity index score, higher Pitt bacteraemia score, and recent hospitalisation were all associated with an increased hazard (daily risk) of in-hospital mortality, and higher Pitt bacteraemia score with a reduced hazard of discharge alive (Table 2).

### 3.3 Excess length of stay

**Fig. 1:**
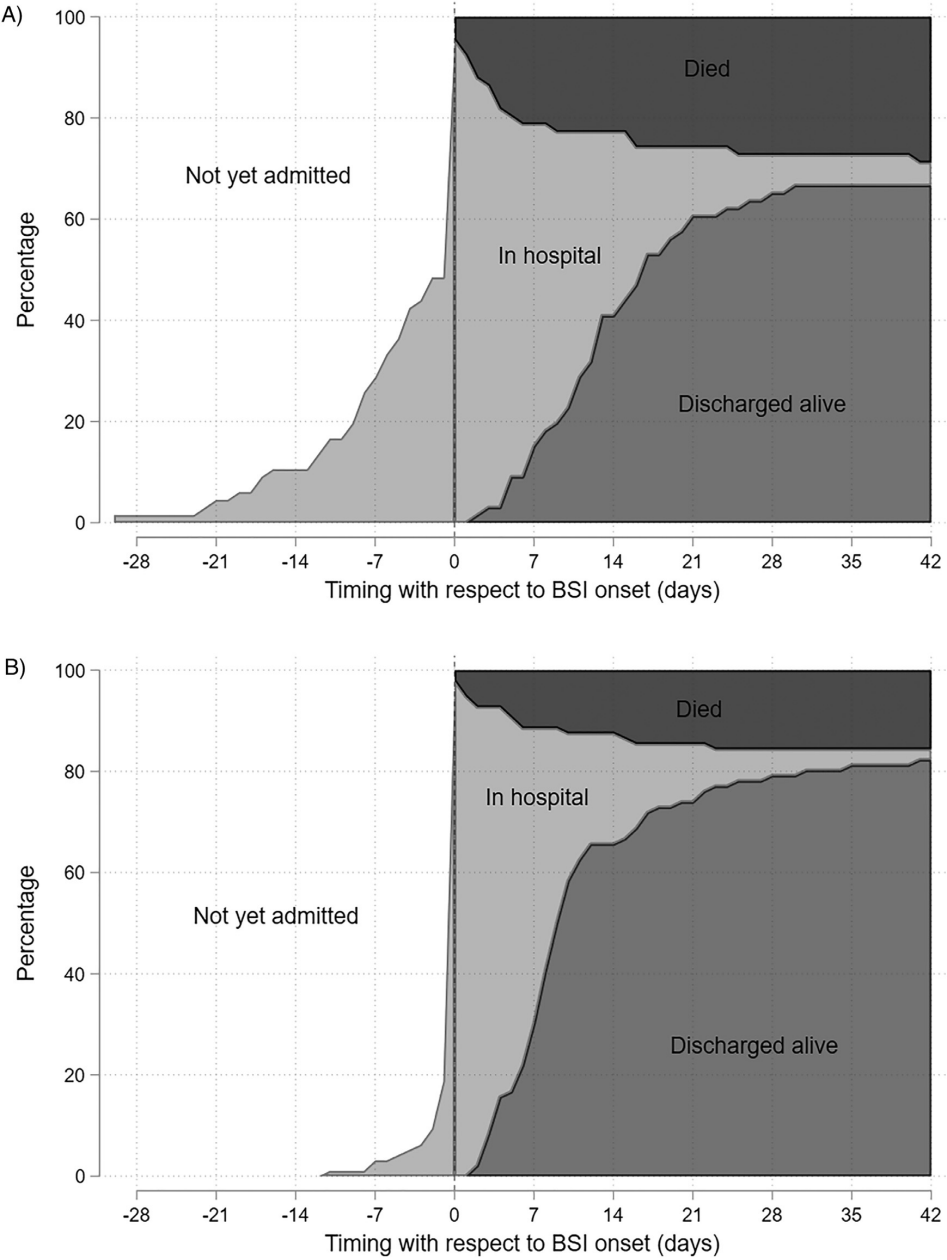
Stacked area charts of patients’ status relative to day of bloodstream infection onset, for 3GC resistant (A) and 3GC susceptible (B) bloodstream infections. Note: Vertical dashed line indicates date of BSI onset (day 0). Patients in hospital (light grey) to the left of the dashed line have not yet experienced BSI, whereas those to the right have. Figures have been restricted to Days -28 to 42 as only one patient changed status before that period (one 3GC-R patient admitted >28 days prior to BSI) and seven patients changed status after that period (five from the 3GC-R group and two from the 3GC-S group).

In the multistate model, 3GC-R was associated with an excess length of hospital stay of 2.6 days (95% CI 2.5–2.8). The proportion of patients occupying each state, according to their day of hospital admission, is presented in Fig. 1.

However, in the composite Cox proportional hazards models (assessing the combined endpoint of either in-hospital mortality or discharge alive), 3GC-R was not associated with the daily risk (hazard) of end of admission in either the univariable (HR 0.89, 95% CI 0.63–1.27) or multivariable (aHR 0.90, 95% CI 0.63–1.30) models.

### 3.4 Bacterial identification and antimicrobial susceptibility testing

Of the 162 episodes of Enterobacterales bacteraemia identified during the study period, 127 (78.4%) had bacterial isolates frozen and shipped to Australia for confirmatory testing. Confirmatory identification using MALDI-TOF MS was performed in Australia on all available isolates, and all were confirmed as Enterobacterales. Two discrepancies were identified at the genus level and five at the species level (Supplementary Table S3). AST was confirmed on all 127 isolates using VITEK-2 except one isolate (0.8%, *M. morganii*) that was incorrectly classified in Suva as 3GC-R instead of 3GC-S.

In the overall cohort, 3GC-R was lower among *E. coli* isolates (34.1%, 29/85) compared to *K. pneumoniae* (47.9%, 23/48) or other Enterobacterales (48.3%, 14/29) (Table 1). AST results for the 127 bacterial isolates with confirmatory testing are summarised in Table 3. Within this subset, all 45 3GC-R isolates remained susceptible to meropenem except for one, a *Providencia rettgeri* possessing a New Delhi metallo-β-lactamase (NDM-1). Among 3GC-R isolates, susceptibility was reduced against other antimicrobials—the next most effective agents were ciprofloxacin and gentamicin (both 15/48, 31.2%) and trimethoprim-sulfamethoxazole (14/48, 29.1%); however, there was variability between species. All 3GC-S isolates were susceptible to meropenem, and overall susceptibility rates >90% were also noted for ciprofloxacin (78/79, 98.7%) and gentamicin (74/79, 93.7%). Including the 35 further isolates that only had AST performed in Suva—where a combination of VITEK and disc diffusion testing was used—did not significantly change the AST results presented above or in Table 3.

**Table 3:** Antimicrobial susceptibility of isolates with confirmatory testing (n = 127)

	3GC resistant (n = 48)			3GC susceptible (n = 79)		
	*E. coli* (n = 22)	*K. pneumoniae* (n = 17)	Other (n = 9)	*E. coli* (n = 43)	*K. pneumoniae* (n = 23)	Other (n = 13)
Ceftriaxone	0 (0%)	0 (0%)	0 (0%)^a^	43 (100%)	23 (100%)	13 (100%)
Amoxycillin-clavulanate	5 (22.7%)	5 (29.4%)	0 (0%)	40 (93%)	23 (100%)	7 (53.8%)
Ciprofloxacin	6 (27.3%)	5 (29.4%)	4 (44.4%)	42 (97.7%)	23 (100%)	13 (100%)
Gentamicin	6 (27.3%)	5 (29.4%)	4 (44.4%)	41 (95.3%)	23 (100%)	10 (76.9%)
Meropenem	22 (100%)	17 (100%)	8 (88.9%)	43 (100%)	23 (100%)	13 (100%)
TMP-SMX	3 (13.6%)	7 (41.2%)	4 (44.4%)	29 (67.4%)	22 (95.7%)	11 (84.6%)

TMP-SMX, trimethoprim-sulfamethoxazole.

a Includes six isolates (three *E. cloacae*, two *K. aerogenes*, and one *E. bugandensis*) that tested phenotypically susceptible to ceftriaxone but were reassigned as 3GC-R because of expression of chromosomal AmpC [13].

### 3.5 Antimicrobial treatment

The most common agents prescribed as part of patients’ initial antibiotic therapy—a time when culture and susceptibility results typically remained unknown—were gentamicin (82/162 patients, 50.6%), cloxacillin (76/162, 46.9%), ceftriaxone (68/162, 42.0%), and ampicillin (51/162, 31.5%). Just two patients received meropenem as part of their initial treatment. Patients with 3GC-R BSIs were less likely to receive active initial therapy (13/66, 19.7%) compared to those with susceptible infections (79/96, 82.3%). Active initial therapy was associated with a significantly reduced hazard of in-hospital mortality on univariable analysis (aHR 0.49, 95% CI 0.24–0.99), but this was no longer significant after adjusting for Pitt bacteraemia score, Charlson comorbidity index score, and recent hospitalisation on multivariable analysis (aHR 0.63, 95% CI 0.28–1.38) (Supplementary Table S4).

### 3.6 Molecular epidemiology

A representative subset of 68 bacterial isolates underwent WGS. Among 29 *E. coli* isolates, we found 13 sequence types (STs) (Supplementary Table S5). Two sequence types, ST131 (n = 7) and ST69 (n = 5), made up more than 40% of the samples. All ST131 were 3GC-R, and only one remained susceptible to ciprofloxacin. These were seen across a mixture of inpatient wards (n = 3), ICU (n = 1), the emergency department (n = 2), and outpatients (n = 1). On pairwise analysis of all ST131 isolates, no evidence of strain transmission was found. All observed ST69 *E. coli* isolates were 3GC-S and were collected from patients who were either in the emergency department (n = 2) or were outpatients (n = 3). The most frequently detected 3GC-R genes among *E. coli* were CTX-M-15 (n = 7), OXA-1 (n = 7), and CTX-M-27 (n = 4) (Supplementary Table S6). Of the 32 *K. pneumoniae* isolates sequenced, we found a variety of STs (n = 21), each of which consisted of three or fewer genomes, with no dominant ST observed. The most frequently detected 3GC-R genes among *K. pneumoniae* were CTX-M-15 (n = 14) and OXA-1 (n = 5).

## 4 Discussion

Our analysis of Enterobacterales bloodstream infections in Fiji demonstrated an unexpectedly high rate of third-generation cephalosporin resistance, as well as high all-cause mortality (regardless of resistance profile) of nearly 1 in 4 patients (22.2%). In this setting, we were unable to detect an association between 3GC-R and mortality after correcting for relevant factors, including patient comorbidities, Pitt bacteraemia score, and length of stay prior to bacteraemia. On multistate modelling, 3GC-R infections were associated with an increased length of hospital stay of 2.6 days; however, this finding was not supported by Cox proportional hazards modelling.

Over the study period, 40.7% of Enterobacterales BSIs at CWMH were 3GC-R, approximately double the previous estimate of 12% to 25% among mixed sample types reported by Fiji to WHO in 2014 [22]. This difference could be partly explained by the later period of our study, with AMR rising globally among Gram-negative pathogens over time [23]. Our reported prevalence of 3GC-R is far higher than recent estimates from other Pacific Island countries, with rates between 4% and 16% reported in neighbouring Samoa, Cook Islands, and Kiribati [24], [25], [26]. Fiji also has a high prevalence of 3GC-R compared to high-income countries (HICs) in the Asia-Pacific region, with just 13.4% (*E. coli*) and 8.6% (*K. pneumoniae*) 3GC-R reported among BSIs in Australia in 2020 [27], and 12.6% (all Enterobacterales) among hospitalised patients in the United States in 2017 [28]. However, resistance remains far lower than that seen in regional AMR ‘hot-spots’ such as India, where up to 80% (*E. coli*) or 90% (*K. pneumoniae*) of bloodstream isolates are 3GC-R [29].

We observed an all-cause in-hospital mortality rate of 16.7% among 3GC-S infections vs. 30.3% among 3GC-R infections, almost double the in-hospital mortality rates (10.1% vs. 16.1%) reported from an equivalent multi-centre study in Europe [30]. Our findings are more comparable to other estimates from LMICs, including 31%–34% vs. 37%–43% from a multi-centre African study [31], and 12% vs. 29% from Thailand [32]. While AMR remains important as a potential and growing contributor to health inequality between HICs and LMICs [4], it is noteworthy that the mortality even for susceptible BSIs in LMICs remains worse than equivalent resistant infections in HICs. While the high prevalence of AMR in Fiji may suggest a need to curb antimicrobial excess, the high mortality rate is a reminder of the enduring importance of antimicrobial access for those with severe infections*.* Efforts to reduce global health inequalities in sepsis outcomes must also focus on non-AMR factors, such as early recognition of at-risk patients, early source control when relevant, early antibiotic therapy, and careful fluid therapy [33].

Despite the difference in all-cause mortality between the two groups, we did not identify an association between AMR and the hazard of in-hospital mortality after correcting for relevant confounders (aHR 1.13, 95% CI 0.51–2.53). We note a recent systematic review by Shamsrizi et al. suggesting that ESBL-producing Enterobacterales BSIs have an increased risk of death (RR 1.70, 95% CI 1.52–1.90); however, in contrast to our research the studies included in this review were largely from HICs, and mostly provided unadjusted estimates [34]. Our findings are more consistent with the results of two recent studies from LMICs. A 2020 matched parallel cohort study across six African hospitals, analysing more than 2500 *E. coli* and *K. pneumoniae* BSIs, found no relationship between 3GC-R and mortality when compared to 3GC-S infections [31]. A 2016 Thai study of more than 6000 BSIs reported multi-drug resistance was associated with increased mortality only for *E. coli*, but not *K. pneumoniae*; however, this study made no adjustment for disease severity or comorbidities and so may represent an overestimate [35].

There are several possible reasons contributing to the absence of an association between 3GC-R and mortality in our study. First, consistent with many of the studies above, no true relationship may exist once patient factors are corrected for. Second, a small true mortality difference may exist that our study was unable to detect. Although we exceeded our target study size, we observed a lower mortality rate in the 3GC-R group than we had anticipated from the literature (30%, vs. an expected 45%), and there is uncertainty around our point estimate. This lower-than-expected mortality rate from 3GC-R infections may be because of CWMH being one of the few healthcare facilities in Fiji with a microbiology laboratory on-site, meaning that clinicians have relatively swift access to culture and susceptibility results. It is possible that if the study was conducted in nontertiary healthcare settings in Fiji—where there may be longer delays in detecting AMR and initiating effective antimicrobials—3CG-R would have had a larger effect on mortality. Finally, while AMR itself may not have been associated with an increased mortality hazard (i.e., daily risk), by prolonging patients’ length of hospital stay it could increase their cumulative risk of dying in hospital.

Our two models assessing the effect of 3GC-R on excess length of stay reached slightly different conclusions. The multistate model, providing output in the more easily interpreted format of ‘excess days’, found 3GC-R BSIs were associated with a significantly increased length of stay of 2.6 days (95% CI 2.5–2.8). In contrast, the Cox proportional hazards model estimates the effect of 3GC-R on the daily risk (hazard) of an all-cause end-of-stay endpoint (either death or discharge alive). Using this method, we did not detect an association between 3GC-R and patients’ daily risk of ending their hospital stay (aHR 0.90, 95% CI 0.63–1.30). It is important to emphasise that while the multistate model provides a cumulative estimate for excess length of stay, the Cox model is estimating the daily effect (hazard) of 3GC-R on length of stay. This is one potential explanation for the discrepancy: that the study was not powered to detect a small change in daily hazard (Cox model) but could detect the larger overall effect of 3GC-R on excess length of stay (multistate model). Second, while both models account for time-dependent bias, only the Cox model was adjusted for confounders. Although it is possible that the output from the multistate model is biased because of confounding, previous studies have suggested that time-dependent bias is the most important factor in length-of-stay analysis [36,37]. Furthermore, to our knowledge there is only one study that has used an adjusted multistate model (using pseudo-value regression) to estimate the effect of antimicrobial resistance on excess length of stay [30]. In that case, the adjusted excess length of stay estimate was actually greater than the unadjusted estimate.

There are a number of key strengths to this study. These include prospective data collection with detailed information including severity of illness at bacteraemia (absent from many large retrospective studies), correction for time-dependent bias when assessing excess length of stay, confirmatory microbiological testing of most isolates, and the inclusion of all age groups. Additionally, although the research was conducted during the COVID-19 pandemic, throughout the study period there was no community transmission of SARS-CoV-2 in Fiji and very few restrictions beyond the closure of the international border. Limitations of this study include it potentially being underpowered to detect a small true difference in mortality, and reduced generalisability as a single-centre study from a tertiary centre. For pragmatic reasons, consistent with the WHO GLASS protocol, only in-hospital mortality was assessed; however, this may have missed an association between AMR and increased mortality at later timepoints. Finally, we did not include an uninfected comparison group, so we cannot assess the effect of BSI on mortality and hospital length of stay compared to no infection.

## 5 Conclusion

Our analysis of Enterobacterales BSIs at Fiji's largest hospital demonstrated a high prevalence of 3GC-R and also a high overall mortality rate, regardless of susceptibility profile. 3GC-R infections were associated with an increased length of hospital stay on multi-state modelling, but we were unable to detect increased in-hospital mortality after correcting for relevant factors. Accurate estimates of the true burden of AMR are important, especially from LMICs, which are under-represented in the literature. Such knowledge can inform policy decisions, guide allocation of limited resources, and assist the evaluation of future interventions to address AMR.

## Appendix Supplementary materials

Image, application 1
