# Supplementary material for: Attributable mortality and excess length of stay associated with third-generation cephalosporin-resistant Enterobacterales bloodstream infections: a prospective cohort study in Suva, Fiji
Source: J Glob Antimicrob Resist. 2022 Sep;30:286–93. doi: 10.1016/j.jgar.2022.06.016 (PMC9452645; doi:10.1016/j.jgar.2022.06.016)
Supplement: Supplementary file 1 [file mmc1.docx]

**Supplementary Appendix**

**Supplement to: Loftus, Young-Sharma et al. Attributable Mortality and Excess Length of Stay associated with Third-Generation Cephalosporin Resistant Enterobacterales Bloodstream Infections – a prospective cohort study in Suva, Fiji**

**Contents**

**Supplementary Table S1……………………………………………………………………………… 2**

Differences between data collection in our study versus WHO GLASS Method for Estimating Attributable Mortality of AMR Bloodstream Infections

**Supplementary Figure S1……………………………………………………………………………. 3**

Directed Acyclic Graph (DAG) process

**Supplementary Table S2……………………………………………………………………………… 5**

Comparison of Adult and Paediatric cases

**Supplementary Table S3……………………………………………………………………………… 7**

List of discrepancies in organism identification between Suva and Melbourne laboratories

**Supplementary Table S4** **…………………………………………………………………………….. 7**

Effect of active initial therapy and patient factors on in-hospital mortality and discharge alive, using Cox proportional hazards models

**Supplementary Table S5** **…………………………………………………………………………….. 8**

Sequence type (ST) of isolates that underwent whole genome sequencing

**Supplementary Table S6** **…………………………………………………………………………….. 9**

Third-generational cephalosporin resistance genes detected on whole genome sequencing

**References** **…………………….………………………………………………………………………….. 9**

**Supplementary Appendix Table S1: Differences between data collection in our study versus WHO GLASS Method for Estimating Attributable Mortality of AMR Bloodstream Infections ^1^**

| **Differences to Core variables from GLASS Method** | | | |
| --- | --- | --- | --- |
| **GLASS Variable(s)** | **WHO GLASS** | **CWMH study** | **Rationale** |
| **Admission Ward**  (“*Responsible medical specialty at time of Admission”*) | 12 coded values including Emergency Department (ED) | Shorter list of coded values with specialty bed cards, no ED option | No ED short stay unit at CWMH, all admitted patients come under a specialty unit |
| **Empirical Treatment** | Antibiotic(s) patient received to treat BSI before microbiological results available | Antibiotic(s) patient received on Days 0-2 of positive blood culture. Defined as “Initial Treatment” | Simplification for data collection at CWMH, as very hard to determine from the notes precisely when microbiology results were available to the treating team |
| **Date Empirical; Empirical End Date** | Date empirical treatment was administered/ stopped | Not collected | See rationale for Empirical Treatment above |
|  | | | |
| **Differences to Optional variables from GLASS Method** | | | |
| **GLASS Variable(s)** | **WHO GLASS** | **CWMH study** | **Rationale** |
| **Transfer From Hospital; Transfer From Non Hospital Facility** | Two separate Boolean Yes/No questions | All captured within ‘**Source of Admission**’ question with drop-down options | Streamlining data collection process for CWMH |
| **Antibiotics YN;**  **Antibiotics YN Name;**  **Antibiotics Before Culture;**  **Antibiotics Before Culture Name** | These four questions focus on any antibiotics received between admission and index culture, separated into those that were given more/less than 48hrs prior to positive culture. | Only asking about:  1) Antibiotics received Days 0-2 from index culture (Initial Treatment), and  2) Any additional antibiotics received between admission and index culture (Other antibiotics) | Have streamlined data collection process at CWMH to reduce data collection time and assist recruitment of more patients. |
| **BSI Source** | One question only, Primary and Central Line infections not differentiated (both as ‘Primary’). Limited options for Secondary foci. | Based on PANORAMA study,^2^ one initial question specifying Primary vs Central Line vs Secondary, and then follow up question (if Secondary) about source of infection | Allows better differentiation of Primary vs Central Line infections, and also provides more detailed options for sources of Secondary infections. |
| (**Infection Type**) – not a specific GLASS variable | GLASS protocol refers to classifying infections as Community-origin vs Hospital-origin. No direct question for this; presumably cases classified by comparing date of admission against date of positive blood culture. | Explicit question in survey. Three options of community-acquired, hospital-acquired and also healthcare-associated community-onset^3^ | Collects this important variable during data entry, provides third category to provide more detailed information. |

**Supplementary Appendix Figure S1 - Directed Acyclic Graph (DAG) process**

We created two Directed Acyclic Graphs (DAGs) to identify minimal sufficient adjustment sets that would be required to estimate the adjusted effect of antimicrobial resistance on 1) in-hospital mortality and 2) hospital length of stay. DAGs were created using DAGitty at http://www.dagitty.net.^4^

Both adjustment sets contained similar variables. Age-adjusted Charlson score, ICU admission and long length of stay prior to bloodstream infection were present in both; for in-hospital mortality, recent hospitalisation (within the last 90 days) was also included. Due to strong correlation between the Charlson score and ICU admission (p = 0.0001), Pitt bacteraemia score was used in place of ICU admission – both are markers of patients’ disease severity at the time of bacteraemia. Receipt of effective initial antibiotics was not included in the multivariable model of as this was on the causal pathway between AMR and both death or discharge.

Of note, organism type was recognised as a potentially relevant factor given some literature suggesting pathogen-related factors can influence mortality even after correction for patient-related factors.^5^ As an exploratory analysis, organism type (*E. coli* vs *K. pneumoniae* vs other) was added to each multivariable model, however this did not improve model fit.

**FIGURE S1A – DAG for impact of Antimicrobial Resistance on In-Hospital Mortality (or the competing outcome of Discharge Alive)**


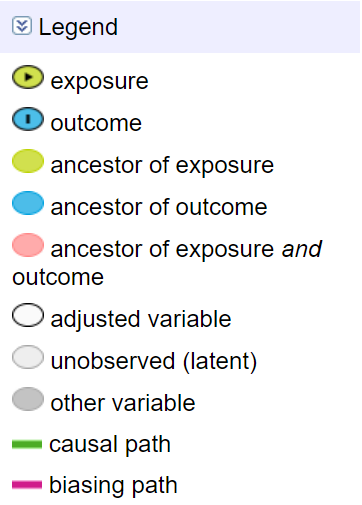

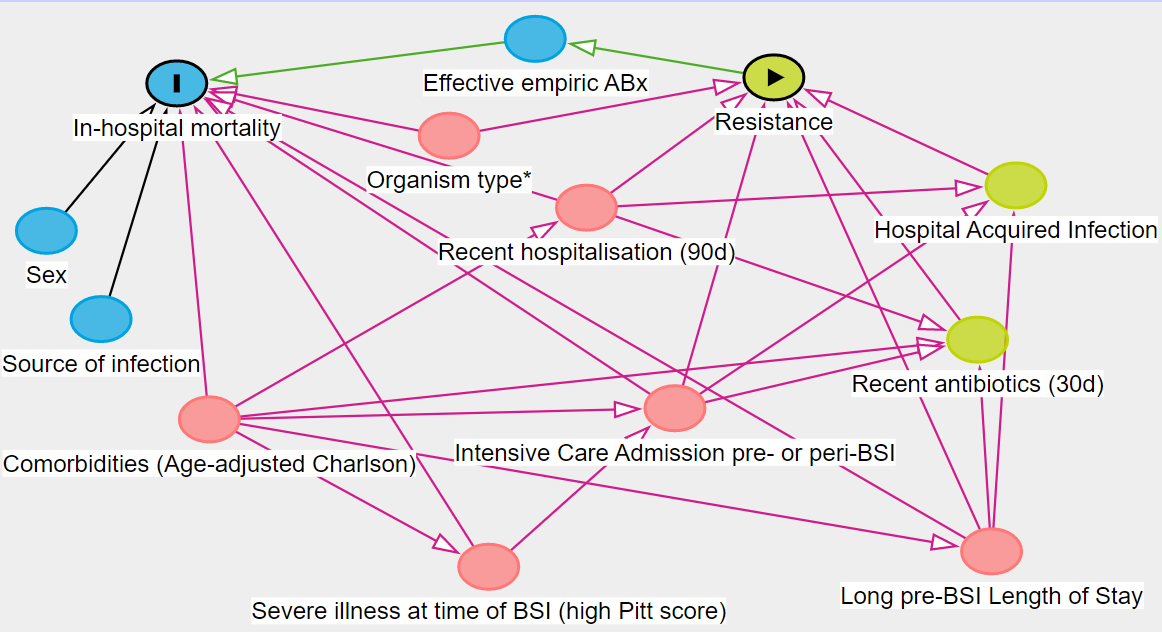


* Organism type included in exploratory analysis to assess improvement in model fit

**FIGURE S1B – DAG for impact of Antimicrobial Resistance on Hospital Length of Stay**


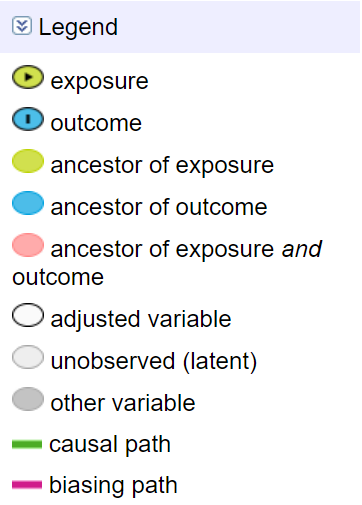

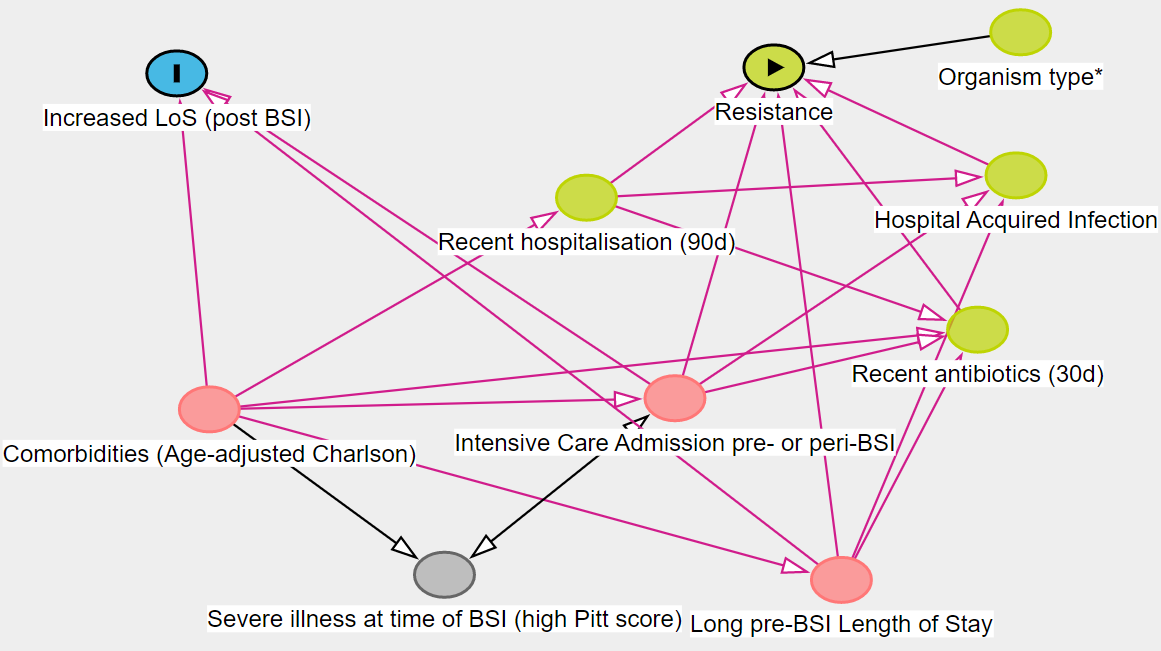


* Organism type included in exploratory analysis to assess improvement in model fit

**Supplementary Appendix Table S2: Comparison of Adult and Paediatric cases**

|  | **Overall** | **Adults**  **(≥18 years)** | **Paediatrics**  **(<18 years)** |
| --- | --- | --- | --- |
| n | 162 | 142 | 20 |
| **Demographics** | | | |
| 3GC-R, n (%) | 66 (40.7%) | 58 (40.8%) | 8 (40.0%) |
| Male, n (%) | 71 (43.8%) | 60 (42.3%) | 11 (55.0%) |
| Median age (IQR) | 55.4 (38.1, 67.7) | 58.5 (48.3, 70.0) | 0.1 (0.0, 0.5) |
| Source of admission, n (%) |  |  |  |
| Home | 114 (70.8%) | 104 (73.8%) | 10 (50.0%) |
| Transfer from another healthcare facility | 39 (24.2%) | 37 (26.2%) | 2 (10.0%) |
| Birth | 8 (5.0%) | 0 (0.0%) | 8 (40.0%) |
| **Acquisition of BSI** | | | |
| Community acquired | 107 (66.5%) | 96 (68.1%) | 11 (55.0%) |
| Hospital acquired | 39 (24.2%) | 31 (22.0%) | 8 (40.0%) |
| Healthcare associated community onset | 15 (9.3%) | 14 (9.9%) | 1 (5.0%) |
| **Healthcare exposures** | | | |
| Antibiotics in 30 days prior, n (%) | 58 (36.9%) | 50 (36.2%) | 8 (42.1%) |
| Hospitalised in 90 days prior, n (%) | 36 (22.2%) | 35 (24.6%) | 1 (5.0%) |
| Surgery since admission, n (%) | 17 (10.6%) | 14 (10.0%) | 3 (15.0%) |
| Immunosuppressed, n (%) | 18 (11.3%) | 18 (12.9%) | 0 (0.0%) |
| ICU stay prior to B/C collection, n (%) | 15 (9.3%) | 8 (5.6%) | 7 (35.0%) |
| Median LoS prior to B/C collection (IQR) | 0 (0, 2) | 0 (0, 1) | 0 (0, 4) |
| **Comorbidities** | | | |
| Diabetes, n (%) | 61 (37.7%) | 61 (43.0%) | 0 (0.0%) |
| Renal disease, n (%) | 16 (9.9%) | 16 (11.3%) | 0 (0.0%) |
| Cardiac disease, n (%) | 15 (9.3%) | 15 (10.6%) | 0 (0.0%) |
| **Clinical risk scores** | | | |
| High risk qSOFA score, n (%) | 36 (23.1%) | 35 (24.6%) | 1 (7.1%) |
| Median Charlson Comorbidity Score (IQR) | 2 (0, 4) | 2 (1, 4) | 0 (0, 0) |
| Median Pitt Bacteraemia Score (IQR) | 1 (0, 2) | 1 (0, 2) | 1 (0, 6) |
| **Microbiology** | | | |
| Organism grown |  |  |  |
| *E. coli* | 85 (52.5%) | 73 (51.4%) | 12 (60.0%) |
| *K. pneumoniae* | 48 (29.6%) | 43 (30.3%) | 5 (25.0%) |
| Other | 29 (17.9%) | 26 (18.3%) | 3 (15.0%) |
| Presumed source of infection |  |  |  |
| Primary | 98 (60.5%) | 85 (59.9%) | 13 (65.0%) |
| Central line associated | 7 (4.3%) | 6 (4.2%) | 1 (5.0%) |
| Secondary | 57 (35.2%) | 51 (35.9%) | 6 (30.0%) |
| Anatomic origin of infection (for secondary infections only) |  | | |
| Urinary tract infection | 27 (47%) | 26 (51%) | 1 (17%) |
| Skin and soft tissue infection | 12 (21%) | 10 (20%) | 2 (33%) |
| Other | 18 (32%) | 15 (29%) | 3 (50%) |
| **Active empiric antibiotics** | | | |
| Yes | 92 (56.8%) | 80 (56.3%) | 12 (60.0%) |
| No | 61 (37.7%) | 53 (37.3%) | 8 (40.0%) |
| Unknown | 9 (5.6%) | 9 (6.3%) | 0 (0.0%) |
| **Outcomes** | | | |
| Died, n (%) | 36 (22.2%) | 34 (23.9%) | 2 (10.0%) |
| Median overall LoS (IQR) | 10 (6, 17) | 9.5 (6, 15) | 18.5 (8.5, 31.5) |
| Median LoS following BSI (IQR) | 9 (5, 16) | 8.5 (5, 13) | 17 (8.5, 25.5) |

*BC = Blood culture. BSI = Bloodstream infection. ICU = Intensive care unit. IQR = Inter-quartile range. LoS = Length of stay. qSOFA = quick Sequential Organ Failure Assessment.*

**Supplementary Appendix Table S3 – List of discrepancies in organism identification between Suva and Melbourne laboratories**

| **Discrepancy type** | **Suva Result** | **Melbourne Result** |
| --- | --- | --- |
| Genus | *Escherichia coli* | *Citrobacter koseri* |
|  | *Klebsiella pneumoniae* | *Escherichia coli* |
| Species | *Providencia stuartii* | *Providencia rettgeri* |
|  | *Klebsiella pneumoniae* | *Klebsiella variicola* |
|  | *Klebsiella oxytoca* | *Klebsiella pneumoniae* |
|  | *Klebsiella aerogenes* | *Klebsiella pneumoniae* |
|  | *Enterobacter cloacae* | *Enterobacter bugandensis* |

The Melbourne laboratory used MALDI-TOF (Matrix-assisted laser desorption/ionization-time of flight) for species identification.

For any discrepancies the Melbourne result was considered definitive and included in the paper.

**Supplementary Appendix Table S4 – Effect of active initial therapy and patient factors on in-hospital mortality and discharge alive, using Cox proportional hazards models***

|  | **In-hospital mortality**  Hazard ratio (95% CI) | **Discharge alive**  Hazard ratio (95% CI) |
| --- | --- | --- |
| **Univariable model** |  |  |
| Active initial therapy | 0.49 (0.24 – 0.99) | 1.31 (0.88 – 1.95) |
| **Multivariable model** |  |  |
| Active initial therapy | 0.63 (0.28 – 1.38) | 1.14 (0.76 – 1.70) |
| Age-adjusted Charlson comorbidity index | 1.59 (1.34 – 1.88) | 0.93 (0.84 – 1.03) |
| Pitt Bacteraemia Score  0-1  2-3  4+ | Reference  4.23 (1.52 – 11.8)  13.7 (5.41 – 34.9) | Reference  0.79 (0.48 – 1.32)  0.16 (0.07 – 0.39) |
| Recent hospitalisation | 2.05 (1.02 – 4.09) | 0.96 (0.57 – 1.62) |

* Both models included adjustment for pre-BSI hospital length of stay (days)

**Supplementary Appendix Table S5 – Sequence type (ST) of isolates that underwent whole genome sequencing**

| ***Escherichia coli*** | |
| --- | --- |
| **ST** | **Frequency** |
| 131 | 7 |
| 69 | 5 |
| 12 | 3 |
| 10 | 2 |
| 44 | 2 |
| 95 | 2 |
| 349 | 1 |
| 38 | 1 |
| 4481 | 1 |
| 549 | 1 |
| 550 | 1 |
| 58 | 1 |
| 6082 | 1 |
| No ST* | 1 |
| **TOTAL** | **29** |

| ***Klebsiella pneumoniae*** | |
| --- | --- |
| **ST** | **Frequency** |
| No ST* | 4 |
| 14 | 3 |
| 1692 | 3 |
| 17 | 2 |
| 39 | 2 |
| 86 | 2 |
| 101 | 1 |
| 105 | 1 |
| 1805 | 1 |
| 2110 | 1 |
| 23 | 1 |
| 25 | 1 |
| 268 | 1 |
| 334 | 1 |
| 35 | 1 |
| 3529 | 1 |
| 420 | 1 |
| 4283 | 1 |
| 4714 | 1 |
| 477 | 1 |
| 65 | 1 |
| 882 | 1 |
| **TOTAL** | **32** |

* These isolates could not be matched to a recognised sequence type.

A further 7 non-*E. coli* non-*K. pneumoniae* isolates underwent whole genome sequencing however no MLST schema were available for these species. These included 3 *Proteus mirabilis* and 1 each of *Citrobacter koseri, Providencia stuartii, Serratia marcescens* and *Morganella morganii.*

**Supplementary Appendix Table S6 – Third-generational cephalosporin resistance genes detected on whole genome sequencing**

| ***Escherichia coli*** | |
| --- | --- |
| **3GC-R gene** | **Frequency** |
| CTX-M-15 | 7 |
| OXA-1 | 7 |
| CTX-M-27 | 4 |
| OXA-10 | 1 |

| ***Klebsiella pneumoniae*** | |
| --- | --- |
| **3GC-R gene** | **Frequency** |
| CTX-M-15 | 14 |
| OXA-1 | 5 |
| SHV-106 | 3 |
| CTX-M-14 | 1 |
| OXA-10 | 1 |
| SHV-27 | 1 |

**References**

1. World Health Organization. GLASS method for estimating attributable mortality of antimicrobial resistant bloodstream infections. Geneva: World Health Organization,; 2020.

2. Stewardson AJ, Marimuthu K, Sengupta S, Allignol A, El-Bouseary M, Carvalho MJ, et al. Effect of carbapenem resistance on outcomes of bloodstream infection caused by Enterobacteriaceae in low-income and middle-income countries (PANORAMA): a multinational prospective cohort study. Lancet Infect Dis 2019;19(6):601-10.

3. Friedman ND, Kaye KS, Stout JE, McGarry SA, Trivette SL, Briggs JP, et al. Health care--associated bloodstream infections in adults: a reason to change the accepted definition of community-acquired infections. Ann Intern Med 2002;137(10):791-7.

4. Textor J, van der Zander B, Gilthorpe MS, Liskiewicz M, Ellison GT. Robust causal inference using directed acyclic graphs: the R package 'dagitty'. Int J Epidemiol 2016;45(6):1887-94.

5. Scheuerman O, Schechner V, Carmeli Y, Gutiérrez-Gutiérrez B, Calbo E, Almirante B, et al. Comparison of Predictors and Mortality Between Bloodstream Infections Caused by ESBL-Producing Escherichia coli and ESBL-Producing Klebsiella pneumoniae. Infect Control Hosp Epidemiol 2018;39(6):660-7.
